# A Two-Stage Phase 2, Multicenter, Randomized, Double-Blind, Placebo-Controlled Study to Evaluate the Safety and Efficacy of Ec-18 in Altering the Severity and Course of Oral Mucositis Secondary to Chemoradiation Therapy for Squamous Cell Cancers of the Head and Neck

**DOI:** 10.3390/cancers17101663

**Published:** 2025-05-14

**Authors:** Christina Henson, Daniel Clayburgh, Arielle Lee, Deborah Wong, Mahesh Kudrimoti, Steve Lee, Noah Kalman, Krishna Rao, Ki Young Sohn, Jeffrey Crawford, Alessandro Villa, Stephen Sonis

**Affiliations:** 1 The Department of Radiation Oncology, Stephenson Cancer Center, University of Oklahoma, Oklahoma City, OK 73104, USA; 2 The Department of Otolaryngology, Portland VA Medical Center, Portland, OR 97239, USA; daniel.clayburgh1@va.gov; 3 The Department of Medical Oncology, UT Health East Texas HOPE Cancer Center, Tyler, TX 75701, USA; arielle.lee@uttyler.edu; 4 The Department of Hematology Oncology, UCLA Health, Los Angeles, CA 90017, USA; dewong@mednet.ucla.edu; 5 The Department of Radiation Medicine, University of Kentucky HealthCare, Lexington, KY 40536, USA; mkudr0@uky.edu; 6 The Department of Radiation Oncology, Tibor Rubin VA Medical Center, Long Beach, CA 90822, USA; steve.lee5@va.gov; 7 The Department of Radiation Oncology Miami Cancer Institute, Miami, FL 33176, USAalessandro.villa@baptisthealth.net (A.V.); 8 The Department of Hematology Oncology, Southern Illinois University, Springfield, IL 62901, USA; krao@siumed.edu; 9 Enzychem Lifesciences, Englewood, NJ 07632, USA; sky@enzychem.com; 10 The Department of Medicine, Duke Cancer Institute, Durham, NC 27710, USA; jeffrey.a.crawford@duke.edu; 11 Primary Endpoint Solutions, Waltham, MA 02451, USA; ssonis@bwh.harvard.edu

**Keywords:** oral mucositis, efficacy, treatment, EC-18

## Abstract

Background: Oral mucositis (OM) remains a significant toxicity of concomitant chemoradiation (CRT) for head and neck cancer (HNC). This trial assessed the safety and efficacy of EC-18, an innate immune response mitigator, in attenuating severe OM (SOM) in HNC patients being treated with CRT. Methods: This was a two-stage, Phase 2, randomized, double-blind, placebo-controlled, multi-institutional trial. Stage 1 consisted of a blinded parallel group dose-finding safety and tolerability study of 24 subjects in four equally sized groups of EC-18 (500 mg, 1000 mg, or 2000 mg or placebo). Stage 2 randomized subjects (1:1) to receive placebo or 2000 mg of EC-18. Twice-daily dosing was carried out from the first to the last day of radiation (LDRT). Patients were assessed twice weekly. OM scores were assigned centrally using WHO criteria. Adverse events were reported using NCI-CTCAE v4.0 criteria. Tumor response was reported up to 12 months following the LDRT. Results: Among patients who received a cumulative radiation dose of at least 55 Gy, at least 80% were compliant with the study’s drug dosing during the first 28 days of treatment and continued to use the study drug for more than 4 weeks. EC-18 effectively reduced the duration, onset, and incidence of SOM compared to placebo. Opioid use was delayed in EC-18-treated patients. Efficacy was associated with weekly cisplatin use and HPV positivity. No significant differences in AEs were observed between study cohorts. Conclusions: EC-18 administered orally may be a safe and effective CRT-associated SOM intervention in patients with HNC.

## Simple Summary

Oral mucositis is a serious and common toxicity of radiation therapy to the head and neck. Despite the severity of its impact on patients, there is no approved drug to prevent or treat severe OM. The innate immune response is a critical initiator of radiation-induced tissue injury. This Phase 2 clinical trial tested the ability of EC-18, an innate immune response mitigator, given as a capsule twice daily to patients undergoing radiation therapy for head and neck cancer, to safely and effectively attenuate the course and severity of mucositis. The results of this randomized, placebo-controlled, blinded, multi-institutional study suggest that EC-18 is safe and effectively mitigates severe OM when taken as directed.

## 1. Introduction

Oral mucositis (OM) remains one of the most debilitating toxicities in patients with head and neck cancer (HNC) receiving concurrent chemoradiation therapy [1,2,3]. In particular, severe oral mucositis (SOM) stands out for its extensive and deep ulcerations of the oral mucosa. The resulting pain is profoundly debilitating, disrupting basic functions like eating, sleeping, and speaking, and is associated with increased visits to the emergency room and hospitalization [4,5,6]. Also, significantly, the intolerable nature of SOM can lead to radiation treatment breaks, which compromise response to treatment and patient outcomes [7,8].

The treatment landscape for SOM (defined as World Health Organization [WHO] Grades 3 or 4) remains limited and is mostly focused on symptom management through the use of topical agents and systemic analgesics [9,10]. As of today, no FDA-approved medications exist to effectively reduce the duration, frequency, or severity of SOM in patients with HNC. This underscores a significant gap in addressing the clinical needs of SOM management, emphasizing the urgent necessity for effective interventions to alleviate the impact of SOM.

EC-18 (1-Palmitoyl-2-linoleoyl-3-acetyl-rac-glycerol [PLAG] is a synthesized version of a naturally occurring small molecule originally derived from Sika deer antlers that is being developed as a potential intervention for SOM in HNC patients [11]. The mechanistic rationale for EC-18 as an SOM mitigator lies in its activities in blunting the innate immune response, a critical element in the initiation of the biological cascade which characterizes radiation-induced tissue injury [12,13]. EC-18 acts as a pattern recognition receptor (PRR) endocytic trafficking accelerator. By eliminating dangerous and foreign signals, such as damage-associated molecular patterns (DAMPs) and pathogen-associated molecular patterns (PAMPs) [14,15], EC-18 downregulates pro-inflammatory chemokines (TNF-α, IL-1β, IL-6) and cytokines (CXCL1/2/8), interrupting necroptosis and preventing collateral damage to neighboring cells [11,14,16], as noted in preclinical murine studies [14]. EC-18 was granted Fast Track Designation by the U.S. Food and Drug Administration in March 2018.

We now report on the safety, tolerability, and efficacy results of a two-stage Phase 2 trial of orally administered EC-18, specifically examining its proficiency in reducing the duration, incidence, and trajectory of SOM in HNC patients receiving chemoradiation therapy.

## 2. Materials and Methods

### 2.1. Study Design

This was a 2-stage, Phase 2, multi-institutional (*n* = 21), randomized, double-blind, placebo-controlled study sponsored by and financially supported by Enzychem Lifesciences. The trial was reviewed and approved by institutional IRBs and registered on ClinicalTrials.gov (NCT03200340). Written informed consent was obtained from study participants. This study was conducted in accordance with the Declaration of Helsinki.

### 2.2. Study Participants

The study participants were adults (>18 years) with histologically confirmed squamous cell carcinomas of the oral cavity, oropharynx, hypopharynx, and nasopharynx who were scheduled to receive standard fractionated daily IMRT of 2.0–2.2 Gy for a minimum cumulative dose of 60 Gy to 72 Gy with concomitant cisplatin administered either weekly or tri-weekly using standard dosing regimens (30–40 mg/m^2^ or 80–100 mg/m^2^, respectively). The planned radiation fields included at least two sites at risk of SOM (buccal mucosa, floor of mouth, lateral/ventral tongue, tonsillar pillars, or soft palate), with both sites planned to receive a cumulative radiation dose of at least 55 Gy. Subjects were required to be able to eat a normal diet at the time of study entry.

### 2.3. Treatment and Assessments

**Figure 1:**
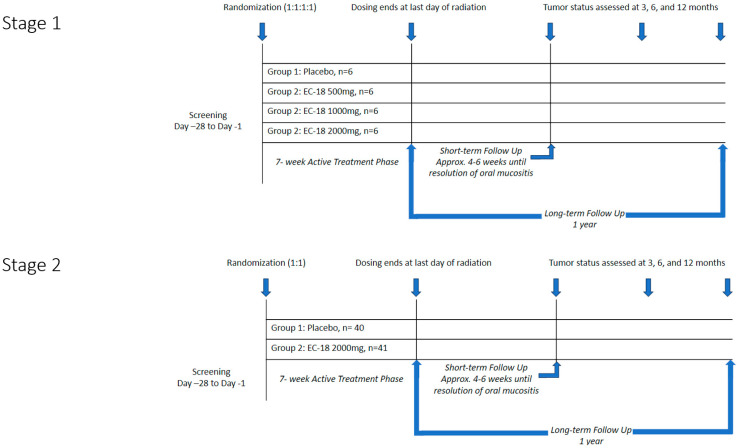
Study design.

A dose escalation study was conducted previously as a Phase 1 clinical trial, and the findings have been summarized on ClinicalTrials.gov under the identifier NCT02532712 [16]. Doses ranging from 500 to 4000 mg confirmed efficacy and safety, with optimal efficacy observed in the 2000 mg group. No serious adverse events were reported in any dose group. The trial was conducted in two stages (Figure 1). Stage 1 consisted of a blinded, parallel group, dose-finding design in which 24 subjects were randomized into four equally sized groups to compare the safety and tolerability of three doses of EC-18 (500 mg, 1000 mg, or 2000 mg) vs. placebo. Results were assessed by an independent Data Safety Management Committee.

**Figure 2:**
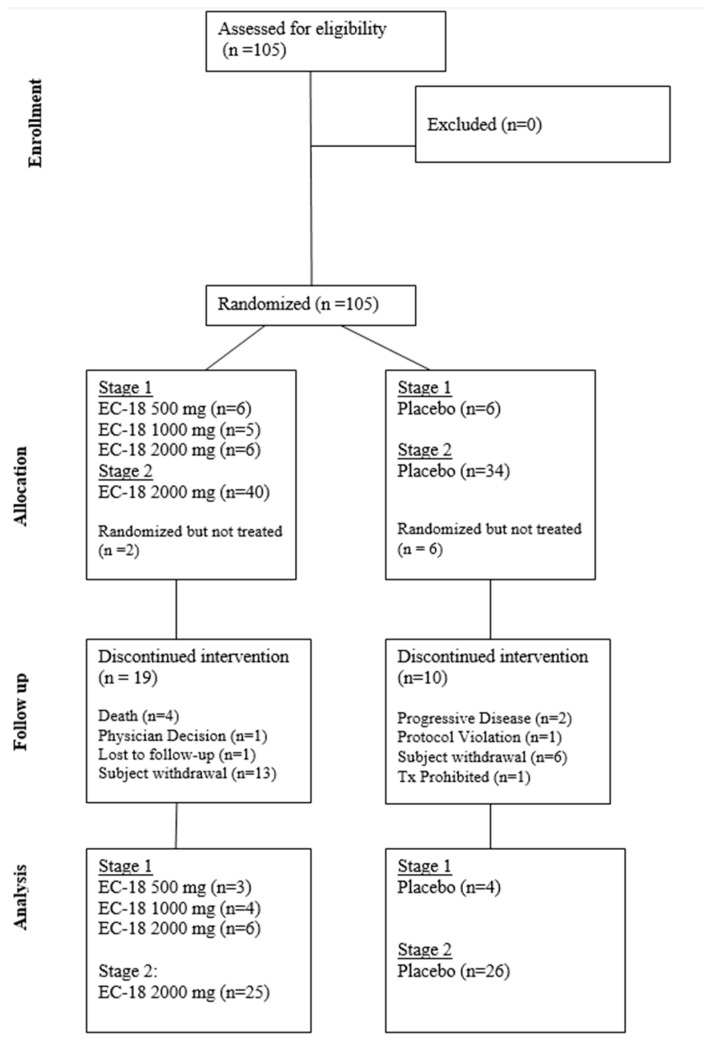
Consort diagram.

Stage 2 evaluated both the safety and efficacy of the highest acceptable dose of EC-18 based on the Stage 1 results (2000 mg per day). Eighty-one subjects were randomized in a 1:1 scheme to receive either placebo or EC-18 (Figure 2). The study drug (EC-18 1000 mg or placebo) was administered twice daily (2000 mg per day) within 30 min of the patient’s first or last meal, beginning on the first day of radiation and continuing until the last day of radiation.

OM severity was assessed twice a week by trained investigator–evaluators using functional, subjective, and objective criteria to secure data that were centrally scored using criteria that informed assignment of mucositis scores using World Health Organization (WHO) criteria. WHO scoring was selected based on its consistency, universality, regulatory acceptance, and practicality and ease of use. Severe OM as defined by WHO criteria tracks well with other established scoring paradigms, such as those noted in CTCAE and RTOG [17]. Patient-reported outcomes (PROs) were assessed on each day of radiation using the Oral Mucositis Daily Questionnaire (OMDQ) and once weekly using the Functional Assessment of Cancer Therapy for Subjects with Head & Neck Cancer (FACT-HN) until all clinical evidence of ulcerative mucositis had resolved (WHO score < 2) (i.e., until the end of STFU). The short-term follow-up (STFU) began the day after the last day of radiation and continued for approximately 4–6 weeks until clinical and symptomatic signs of OM had resolved. The long-term follow-up (LTFU) extended for 12 months following the last dose of radiation and assessed response to CRT using the Response Evaluation Criteria in Solid Tumors (RECIST) criteria. Tumor status was evaluated approximately 3 months, 6 months, and 12 months following the last dose of radiation therapy.

Safety was evaluated every 2 weeks until 30 days after the last dosing of Stage 1 and throughout the treatment. Safety endpoints included the incidence of adverse events, based on NCI-CTCAE v4.0 criteria, as well as serious adverse events (SAEs) and clinically significant laboratory anomalies.

### 2.4. Endpoints and Statistical Methods

Safety data including SAEs, treatment-emergent AEs (TEAEs), vital signs, weight, body mass index (BMI), physical examinations, and clinical laboratory assessments, as well as tumor response assessments, were summarized by the treatment group using descriptive statistics for all randomized patients (ITT cohort). All AEs were coded by Preferred Term (PT) using the Medical Dictionary for Regulatory Activities (MedDRA) classification dictionary (version 20.0).

Efficacy data were summarized using descriptive statistics by treatment group for each stage of the study, including the number of subjects, median, mean, standard deviation, minimum and maximum values for continuous data, and frequency tables (%) for categorical data. Proportions are presented along with 95% confidence intervals, calculated using Wilson’s score method.

The primary efficacy analysis compared all subjects treated daily with 2000 mg EC-18 (selected dose level for Stage 2) versus all placebo-treated subjects at the conclusion of the STFU. Patients included in this analysis received a cumulative radiation dose of at least 55 Gy, and at least 80% were compliant with the study’s drug dosing during the first 28 days of treatment and continued to use the study drug for more than 4 weeks without experiencing a major protocol deviation (per protocol cohort). RTQA performed by a central monitor confirmed, for each submitted case, that two oral sites were planned to receive a total radiation dose of at least 55 Gy, also confirming that the daily and cumulative prescribed doses were per protocol.

The duration of SOM was calculated as the number of days from the onset of SOM (first time WHO Grade 3 or 4 was observed) to the day when SOM resolved (first time WHO Grade 2 or lower was observed after the last WHO Grade 3 or 4) or until two weeks after the last day of radiation, whichever came first.

Additionally, a secondary analysis was conducted to compare endpoints at the completion of active treatment and through short-term follow-up (STFU) among the ITT non-EC treated patients, patients randomized to the EC-18 arm who were not compliant, and the per protocol placebo patients.

The generalized Cochran–Mantel–Haenszel (CMH) method was used to compare treatment groups. Standardized mid-ranks (also known as modified ridit scores) were used for the test statistic. For the secondary efficacy endpoints (incidence of SOM and incidence of ulcerative OM), the general association form of the stratified CMH test was used for the comparison of proportions. A test of the mean score difference with modified ridit scores was used for continuous variables, including the duration of ulcerative OM, average pain severity scores, and total opioid analgesic use (in morphine equivalents).

Kaplan–Meier methods were used for estimating time to onset of SOM and other time to event endpoints; cumulative distribution curves were compared using log-rank tests, stratified by the study center. Additional sensitivity analyses were performed to support the primary efficacy analysis. To account for multiple comparisons, significance testing of secondary efficacy endpoints employed a hierarchical testing procedure to protect the overall type I error rate of 5% (2-sided).

## 3. Results

### 3.1. Patient Characteristics

Data pertaining to patient demographics, tumor status, and the baseline characteristics of the ITT population are reported in Supplementary Table S1. The patients enrolled (mean age: 61.6 years) were mostly White (89.7% [87/97]), non-Hispanic or Latino (92.8% [90/97]), and male (86.6% [84/97]). The majority of patients had oropharyngeal (73% [71/97]) or oral cavity cancers (22% [21/97]) and were HPV-positive (70% [68/97]). Arms were reasonably matched for demographics, primary tumor location, disease stage, tumor HPV status, and cisplatin regimen.

### 3.2. Compliance

**Figure 3:**
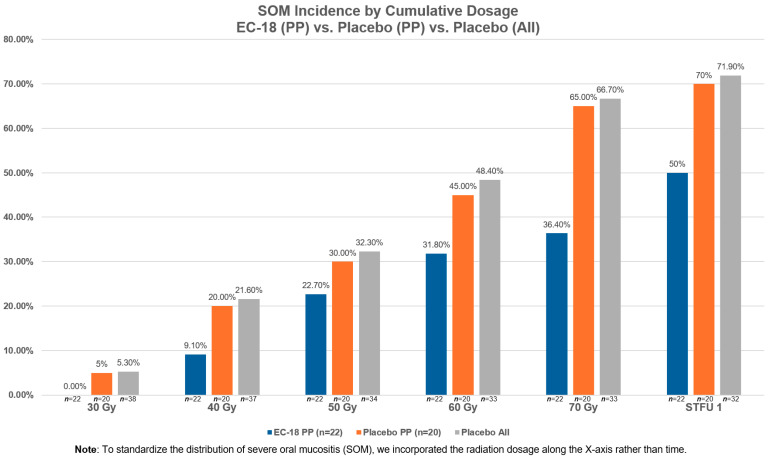
Severe oral mucositis (SOM) incidence by cumulative dosage vs. placebo (PP) vs. placebo (all).

The COVID pandemic affected patients’ overall compliance by impacting three study participants’ ability to (1) receive a cumulative radiation dose of at least 55 Gy; (2) achieve at least 80% compliance with the study’s drug dosing during the first 28 days of treatment and continue to use study drug for more than 4 weeks; and (3) not have a major protocol deviation. The higher incidence of non-compliant patients who received EC-18 and a stomatotoxic dose of radiation compares with 45.5% (10/22) of patients receiving EC-18 and who received treatment per protocol. We further assessed the lack of EC-18 compliance on the severity of OM by evaluating the proportion of SOM by time (each week of treatment represents an additional 10 Gy of radiation) and observed that the lack of EC-18 dosing compliance effectively negated any observed impact of SOM development, compared to the EC-18 patients in the per protocol cohort (Figure 3). Statistical comparison between the non-compliant EC-18-treated patients who received 55 Gy with all placebo patients failed to demonstrate any significant differences in incidence from baseline to the end of treatment (*p* = 0.16) or through STFU (*p* = 0.23). Likewise, the differences in incidence between the non-compliant EC-18-treated patients was statistically unremarkable when compared to the per protocol placebo cohort at either the end of treatment (*p* = 0.18) or through STFU (*p* = 0.29).

We then evaluated the concordance of each group based on factors associated with SOM risk and EC-18 efficacy. No difference was seen in the distribution of cisplatin regimens, which favored weekly dosing, between patients who received EC-18 whether they were compliant or not (64.3% NC vs. 72.7% compliant). Weekly and tri-weekly cisplatin regimens were equally distributed in the per protocol placebo cohort. This observation suggests that the higher incidence of SOM noted in the 55 Gy non-compliant EC-18-treated patients was independent of cisplatin dosing.

The incidence of SOM during the active treatment period was 85.7% (12/14) vs. 65.6% (21/32) vs. 65% (13/20) in the non-per protocol EC-18, all placebo, and per protocol placebo cohorts who had received at least 55 Gy of radiation, respectively (Table 1). When compared to the non-per protocol EC-18 cohort, the all placebo and per protocol placebo cohorts did not demonstrate significant differences in the incidence of SOM (*p* = 0.16 and 0.18, respectively). Similarly, SOM through STFU was noted as 85.7% (12/14), 68.8% (22/32), and 70% (14/20) in the non-per protocol EC-18, all placebo, and per protocol placebo cohorts, respectively. When compared to the non-per protocol EC-18 cohort, the all placebo and per protocol placebo cohorts did not demonstrate significant differences (*p* = 0.23 and 0.29, respectively). On the other hand, for the EC-18 per protocol population, 40.9% (9/22) had SOM during the active treatment period, and 45.5% (10/22) had cases of SOM through STFU which were significantly different when compared to the non-per protocol EC-18 (*p* = 0.008 and 0.02 for two evaluable time points, respectively).

When SOM incidence was compared between the non-compliant EC-18 patients who received at least 55 Gy and the per protocol placebo and EC-18 cohorts with respect to response associated with cisplatin regimen and HPV status, it appeared that the EC-18 non-compliant patient response aligned with the per protocol placebo outcomes.

The Safety Population (ITT) included all patients randomized to treatment who had received at least one dose of the study drug (Stage 1 [23 subjects] and Stage 2 [74 subjects]). A total of 8 out of 97 (8.4%) patients were excluded from the ITT Population due to self-withdrawal before taking the first dose of the study drug.

### 3.3. Efficacy Analysis

#### 3.3.1. Duration of SOM Among All Patients in the per Protocol Cohort

The per protocol population consisted of all subjects who received the study drug for a minimum of 4 weeks, achieved 80% compliance with the study drug up to 28 days after the first dose, and received a cumulative radiation dose of at least 55 Gy to oral and oropharyngeal sites at risk.

**Figure 4:**
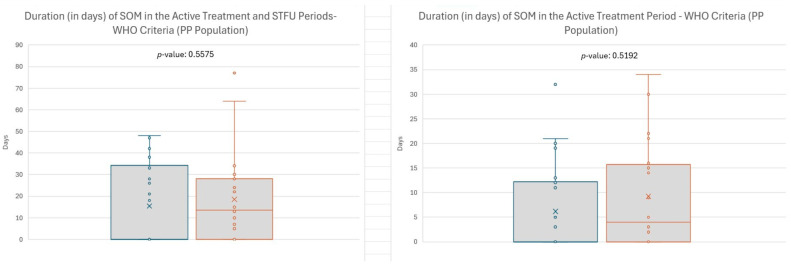
Duration of severe oral mucositis (SOM) during active treatment period. Note: Efficacy analyses include 2000 mg and Placebo subjects from Stage 1 and Stage 2.

Median duration (in days) was calculated for all subjects, including those who never developed SOM (duration = 0 days). EC-18 treatment favorably impacted SOM duration; median duration of SOM (in days) from baseline through the STFU period was shorter in patients treated with EC-18 compared with the placebo group (0 [range: 0–48] vs. 13.5 [range: 0–77]; *p* = 0.5575), as well as from baseline through the active treatment period (0.0 days vs. 4.0 days; *p* = 0.5192) (Figure 4). While differences in SOM duration and incidence did not reach statistical significance, the observed trends support the need for more extensive confirmatory trials.

#### 3.3.2. Incidence of SOM

A non-significant difference in SOM incidence (Chi square) was observed between the per protocol placebo and per protocol EC- 18 cohorts from baseline through STFU (Table 1). Whereas 70% (14/20) of patients in the placebo arm developed SOM between radiation start through short-term follow-up, SOM was only noted in 45.5% (10/22) of the EC-18-treated group (*p* = 0.19). The 70% (14/20) incidence noted in the placebo cohort is consistent with what is expected.

Similarly, among patients in the per protocol population, EC-18 favorably impacted SOM incidence during the active treatment period. Whereas SOM was noted in 65.0% (13/20) of placebo patients, it was noted less (40.9%; *p* = 0.30) in patients treated with EC-18 (Table 1). EC-18 also blunted the SOM trajectory. The proportional incidence of SOM at cumulative radiation doses of 10 Gy (2 Gy fractions were delivered 5 days per week) was favorably attenuated in EC-18-compliant patients vs. placebo controls.

In agreement with current epidemiological trends, the majority of patients (70% [68/97]) who were treated for oropharyngeal cancers were HPV-positive. EC-18 appeared to effectively mitigate SOM incidence in patients with HPV-positive tumors (EC-18 35.3% [6/17] vs. placebo 66.7% [8/12]). The efficacy of EC-18 on SOM in non-oropharyngeal cancer cases was impossible to assess because of the small (*n* = 2) number of patients in the EC-18 arm.

Pre-specified subgroup analysis included a cisplatin regimen. Consistent with current practice trends, the majority of patients received weekly doses of cisplatin. Of patients in this group, EC-18 effectively reduced SOM incidence from 70% (28/40) in the placebo cohort to 37.5% (15/40). In contrast, no difference was seen among patients treated with tri-weekly cisplatin regimens in which 70% (7/10) of placebo patients developed SOM vs. 66.7% (4/6) of EC-18-treated patients.

Regarding G tube use, a total of eleven subjects had G tubes placed. In Stage 1, two patients in the EC-18 500 mg group and two in the EC-18 1000 mg group required G tube placement. In Stage 2, five patients in the study arm and two patients in the placebo arm had G tubes placed. As far as differences in weight loss between the treatment arm and the placebo arm, the mean change in weight between baseline and end of treatment was -8.8% in the EC-18 2000 mg arm versus −11.6% in the placebo arm. This was not statistically significant (*p* = 0.48).

#### 3.3.3. Opioid Analgesic Use to Control Oral Pain

The time to first use of opioid analgesics was delayed for evaluable subjects (those who were not taking opioid analgesics immediately prior to study start or at baseline and those for whom data were available) among the EC-18 group vs. placebo group. Median time to first use of opioids in the EC-18 group was 37 days compared with 26 days in the placebo group (Supplemental Figure S1).

#### 3.3.4. OMDQ and FACT-HN Assessments

OMDQ and FACT-HN did not show improvement in the EC-18 group as compared to the placebo group. The reasons for the disconnect between self-described symptomatic changes, as noted by the above outcomes, and the functional component embedded in WHO score criteria is interesting, somewhat puzzling, and not inconsistent with symptom complexities experienced by patients [18,19,20].

### 3.4. Safety (Adverse Events)

Subjects who were randomized to treatment and received at least one dose of the study drug were included in the Safety Analysis Set and were analyzed according to the treatment they actually received. The same population was used for demographic and baseline data, concomitant medications, and study drug exposure/compliance data. Overall, the incidence of TEAEs across active and placebo cohorts was similar.

A detailed account of TEAEs is presented in Supplementary Table S2. The distribution and frequency of AEs occurring in 10% or more of study participants was similar between the active and control arms and generally attributable to the consequences of the CRT regimens used. Patients in both cohorts were noted to have high frequencies of nausea and other symptomatic AEs of the gastrointestinal tract. The low incidences of stomatitis and oral pain were likely due to inconsistencies in reporting. Since mucositis (stomatitis) was a study endpoint, some investigators did not report it as an independent AE. Laboratory findings associated with increased creatinine levels and leukopenia and thrombocytopenia were similar between study groups and not unexpected given patients’ cancer treatment regimens.

## 4. Discussion

Oral mucositis poses a significant challenge to patients receiving CRT for the treatment of HNC. Despite its significant symptoms, impact on treatment tolerance, and burden on the healthcare system, the only FDA-approved therapeutic options for the prevention or management of OM are limited to palliative devices. Thus, the need for a small molecule or biological that effectively interferes with the pathobiological cascade that leads to mucosal injury is compelling [21].

It is clear that oxidative stress [12,22,23] is a critical initiator of the biological cascade that results in radiation-induced tissue injury. Consequently, interfering with initiation of this cascade as a pharmacologic strategy has a rationale [21,24]. Results of preclinical studies suggest that EC-18’s mechanism of action is well suited for this purpose, as it reduces oxidative stress, activation of both the innate immune response and NF-ƙB, and subsequent pro-inflammatory signals and mediators [14,25,26,27,28].

The current two-stage study was undertaken to identify an optimal safe dose for EC-18 and then to test the safety and efficacy of that dose as an intervention for SOM in patients receiving CRT for HNC. In the first stage of the study, a dose of 2000 mg per day, given as two divided doses, 1000 mg in the morning and evening, was found to be well tolerated and safe. EC-18 was formulated as 500 mg capsules which were taken orally.

A major challenge in and limitation of this study was the timing of recruitment, which overlapped with the Coronavirus disease 2019 (COVID-19) pandemic [29,30,31,32,33]. While the original sample size was powered to provide robust numbers for statistical analysis, the rate of patient recruitment was negatively affected, and restrictions on the ability of site research personnel to perform work on site limited the development of critical patient–research nurse relationships, which impacted accrual. Additionally, the expected compliance rates typically observed with similar formulations for an oral mucositis indication were not seen. Of randomized patients in the test arm, only 44% (41/93) met the requirements to assure comparable SOM risk and adequate study drug dosing to accurately determine possible efficacy. Our observation that the incidence of SOM in non-compliant patients randomized to the active (EC-18) arm was equivalent to both the ITT placebo cohort and the placebo arm of the per protocol population confirmed the importance of dosing compliance. Consequently, any realistic assessment of EC-18’s efficacy mandated a focus on the dosing-compliant (per protocol) population as favorable efficacy signals (overall SOM incidence and proportion of SOM patients by cumulative radiation dose) noted in EC-18-compliant patients was lost when EC-18 non-compliant patients were included. Another factor that potentially impacted compliance was the prohibition of the use of steroids, and as a result, steroids will likely be allowed in future trials. Another potential factor was that it became progressively more difficult for patients to swallow the capsules by mouth throughout the treatment period, and a liquid version will be considered for the next phase of study.

Overall, EC-18-treated patients had a shorter duration of SOM days from baseline through the STFU period compared to the placebo group (0 [range: 0–48] vs. 13.5 days [range: 0–77]; *p* = 0.5575) (Figure 4). Similarly, the incidence of SOM through STFU was 45.5% (10/22) for EC-18-treated patients vs. 70% (14/20) for the placebo group (*p* = 0.1894). EC-18-treated patients reported fewer days in the onset and duration of opioid use compared to patients in the placebo arm (37 days vs. 26 days).

A comparison of SOM incidence between EC-18-compliant patients (45.5% [10/22]) and non-compliant patients (85.7% [12/14]) suggests that there is a rationale for using the per protocol population as the basis for the efficacy analysis.

While EC-18 favorably affected a broad range of efficacy parameters, variations in its effectiveness were seen when patients were stratified by cisplatin regimen, tumor HPV status, and primary tumor site. The impact of EC-18 on SOM incidence was more pronounced in patients receiving weekly cisplatin treatment (EC-18: 37.5%; placebo: 70%, *p* = 0.1), and those with HPV-positive HNC (EC-18: 35.3%; placebo: 66.7%, *p* = 0.09), compared to patients receiving tri-weekly cisplatin dosing or those with HPV-negative tumors (EC-18: 75%; placebo: 71.4%, *p* = 0.9). The distribution of subjects based on HPV status reflects the ratio of OPC cases to non-OPC cancers observed in the study population, aligning generally with the incidence of new OPC cases reported in the United States. In our patient cohort, the incidence of SOM trended lower in the EC-18 2000 mg group compared to the placebo group among patients with OPC (EC-18: 45.0%; placebo: 64.3%, *p* = 0.26) and among subjects with non-OPC (EC-18: 50.0%; placebo: 83.3%, *p* = 0.35), respectively.

The observed association between EC-18 efficacy and cisplatin regimen is interesting but not easily explained. While it is seems clear that the use of CRT confers an increased risk of severe oral mucositis compared to radiation alone, the extent of that risk seems unaffected by cisplatin regimen, as, with occasional exceptions, the literature would suggest that weekly and tri-weekly cisplatin are equally stomatotoxic [34,35,36]. Nonetheless, a close evaluation of data presented in reviews and meta-analysis of the topic provides conflicting results: whereas severe mucositis was more common in weekly cisplatin regimens (51%) compared to tri-weekly regimens (37%) in patients treated postoperatively, the opposite was noted among patients treated with definitive CRT (weekly 25% vs. tri-weekly 42%; *p* = 0.1251). Potential explanations for improved drug efficacy in the setting of weekly cisplatin and HPV positivity include the fact that weekly cisplatin may cause less immunosuppression compared to tri-weekly, and this may allow the drug to work better, as its mechanism is through the innate immune response. Regarding HPV positivity, HPV-associated head and neck cancers tend to occur in younger and healthier patients than HPV-negative ones and have less of an association with tobacco use [37,38]. All of these patient factors could theoretically impact EC-18 efficacy. Additionally, given the mixed data regarding mucositis risk of different cisplatin dosing regimens, if the risk truly is lower with weekly cisplatin, the threshold for effect of a mucositis modulator could be lower.

No safety issues were identified during the trial. The incidence of TEAE was similar across all groups in both stages of the study, and virtually all were attributable to the cancer treatment regimen and not the investigational drug. In Stage 1, 6 serious adverse events (SAEs) were reported in three (50.0%) placebo subjects, and 21 SAEs occurred in eight (23.5%) placebo subjects during Stage 2. The EC-18 2000 mg cohort did not report any SAEs during Stage 1, while 14 were reported across seven (17.5%) patients during Stage 2. All adverse events leading to discontinuation were reported in the EC-18 2000 mg group.

## 5. Conclusions

This study aimed to assess the safety and efficacy of EC-18 in mitigating SOM among patients being treated with concomitant CRT for selected cancers of the head and neck. The data generated suggest that EC-18 was safe and easily administered and yielded encouraging results in patients with HPV-positive tumors who received concomitant CRT with weekly cisplatin dosing. The results justify a larger, more definitive trial to further explore the utility of EC-18 in the management of SOM.

## Figures and Tables

**Table 1:** Incidence of severe oral mucositis (SOM) in EC-18 2000 mg versus placebo cohorts.

Period	STFU				Active Treatment			
Incidence of SOM (Grades 3 and 4) [*n* (%)]	EC-18 2000 mg (*n* = 22)	Placebo (*n*= 20)	Non-PP EC-18 (*n* = 14)	All Placebo (*n* = 32)	EC-18 2000 mg (*n* = 22)	Placebo (*n* = 20)	Non-PP EC-18 (*n* = 14)	All Placebo (*n* = 32)
Incidence of SOM	10 (45.5)	14 (70.0)	12 (85.7)	22 (68.8)	9 (40.9)	13 (65.0)	12 (85.7)	21 (65.6)
95% Confidence Interval ^a^	(26.9, 65.3)	(48.1, 85.5)			(23.3, 61.3)	(43.3, 81.9)		
CMH Test *p*-value ^b^	0.1894				0.2953			

^a^ exact confidence interval was calculated using the Wilson scores method. ^b^ CMH test of general association stratified by (pooled) sites between EC-18 2000 mg and placebo. Note: efficacy analyses include 2000 mg and placebo subjects from Stage 1 and Stage 2.

## Data Availability

Non-confidential or unrestricted data may be available on request from the study sponsor.

## Supplementary Materials

The following supporting information can be downloaded at: https://www.mdpi.com/article/10.3390/cancers17101663/s1. Figure S1: Time to opioid analgesic use (PP Population); Table S1: Demographics Characteristics by Stage; Table S2: Incidence of TEAEs Experienced by ≥10% of subjects treated with 2000 mg/day of EC-18 or Placebo; Table S3: Incidence of AEs experienced by subjects treated with 2000 mg/day of EC-18 or placebo; Table S4: Categorical incidence of SAEs in EC-18 2000 mg vs placebo population; Table S5: ITT duration of SOM during baseline to last dose of RT (BL-LDRT) for all subjects; Table S6: ITT duration of SOM BL-LDRT in subjects who had ≥1 day of SOM; Table S7: Subject compliance.

## Abbreviations

The following abbreviations are used in this manuscript:

AE	Adverse event
CMH	Cochran–Mantel–Haenszel
CRT	Chemoradiation therapy
FACT-HN	Functional Assessment of Cancer Therapy for Subjects with Head & Neck Cancer
HNC	Head and neck cancer
OM	Oral mucositis
OMDQ	Oral Mucositis Daily Questionnaire
LTFU	Long-term follow-up
NC	Non-compliant
PT	Preferred Term
RTQA	Radiation Therapy Quality Assurance
SAE	Severe adverse event
SOM	Severe oral mucositis
STFU	Short-term follow-up
TEAE	Treatment-emergent AEs
WHO	World Health Organization
