# Supplementary material for: A Two-Stage Phase 2, Multicenter, Randomized, Double-Blind, Placebo-Controlled Study to Evaluate the Safety and Efficacy of Ec-18 in Altering the Severity and Course of Oral Mucositis Secondary to Chemoradiation Therapy for Squamous Cell Cancers of the Head and Neck
Source: Cancers (Basel). 2025 May 14;17(10):1663. doi: 10.3390/cancers17101663 (PMC12109969; doi:10.3390/cancers17101663)

Table S1: Demographics Characteristics by Stage

Characteristics	No. (%) of Subjects						Total (N = 97)
	Stage 1				Stage 2		
	Placebo (n = 6)	EC-18 500 mg (n = 6)	EC-18 1000 mg (n = 5)	EC-18 2000 mg (n = 6)	Placebo (n = 34)	EC-18 2000 mg (n = 40)	
**Race**							
White	4 (66.7)	6 (100)	5 (100)	6 (100)	30 (88.2)	36 (90.0)	87 (89.7)
Black or African American	2 (33.3)	0.0	0.0	0.0	1 (2.9)	1 (2.5)	4 (4.2)
Asian	0.0	0.0	0.0	0.0	1 (2.9)	1 (2.5)	2 (2.1)
American Indian or Alaska Native	0.0	0.0	0.0	0.0	1 (2.9)	1 (2.5)	2 (2.1)
Native Hawaiian or other Pacific Islander	0.0	0.0	0.0	0.0	1 (2.9)	1 (2.5)	2 (2.1)
Other	0.0	0.0	0.0	0.0	0.0	0.0	0
**Ethnicity**							
Hispanic or Latino	0	0	0	0	0	2 (5.0)	2 (2.1)
Not Hispanic or Latino	6 (100)	6 (100)	5 (100)	6 (100)	31 (91.2)	36 (90.0)	90 (92.8)
Not Reported	0	0	0	0	1 (2.9)	1 (2.5)	2 (2.1)
Unknown	0	0	0	0	2 (5.9)	1 (2.5)	3 (3.1)
**Sex**							
Female	1 (16.7)	1 (16.7)	1 (20.0)	1 (16.7)	4 (11.8)	5 (12.5)	13 (13.4)
Male	5 (83.3)	5 (83.3)	4 (80.0)	5 (83.3)	30 (88.2)	35 (87.5)	84 (86.6)

**Weight (kg)**							
Mean Weight	96.1	94.7	89.2	86.4	95.5	91.5	92.2
**Height (cm)**							
Mean Height	171.6	171.5	171.7	174.7	175.8	176.1	173.6
**BMI (kg/m^2)**							
Mean BMI	32.8	32.1	30.4	28.3	30.7	29.2	30.6
**Age (Years)**							
Mean Age	59.5	61.3	69.2	61.8	59.3	58.5	61.6
Age range	42-70	58-63	62-79	55-71	40-78	26-78	-
**ECOG**							
0	6 (100)	3 (50)	4 (80)	6 (100)	27 (79)	30 (75)	76 (78)
1	0	3 (50)	1(20)	0	6 (18)	10 (25)	20 (21)
2	0	0	0	0	0	0	0
Unknown	0	0	0	0	1 (3)	0	1 (1)
**Tumor Site**							
Nasopharynx	0	1 (17)	0	0	1 (3)	0	1 (1)
Hypopharynx	0	0	0	0	0	1 (2)	2 (2)
Oropharynx	2 (33)	3 (50)	2 (40)	4 (67)	27 (79)	33 (83)	71 (73)
Oral Cavity	4 (67)	2 (33)	2 (40)	2 (33)	5 (15)	6 (15)	21 (22)
Multiple	0	0	1 (20)	0	0	0	1 (1)
Unknown	0	0	0	0	1 (3)	0	1 (1)
**Tumor, Node, Metastasis Stage**							

0-II	3 (6)	2 (4)	2 (4)	2 (4)	18 (53)	25 (53)	52 (54)
III	1 (5)	3 (15)	2 (10)	3 (15)	7 (21)	4 (10)	20 (20)
IV	2 (8)	1 (4)	1 (4)	1 (4)	9 (26)	11 (27)	25 (26)
**Tumor HPV status**							
Positive	4 (67)	5 (83)	3 (60)	6 (100)	22 (65)	28 (70)	68 (70)
Negative	2 (33)	1 (17)	0	0	9 (26)	9 (23)	21 (22)
Unknown	0	0	2 (40)	0	3 (9)	3 (7)	8 (8)
**Cisplatin Schedule**							
Every 3 weeks (High-dose)	5 (83)	0	2 (40)	1 (17)	14 (41)	12 (30)	34 (35)
Weekly (Low-dose)	1 (17)	6 (100)	3 (60)	5 (83)	20 (59)	28 (70)	63 (65)

Abbreviations: BMI=body mass index; ECOG= Eastern Cooperative Oncology Group; HPV=Human Papillomavirus.

Table S2: Incidence of TEAEs Experienced by ≥10% of subjects treated with 2000 mg/day of EC-18 or Placebo

Preferred Term n (%)	EC-18 2000 mg (N=6 subjects)		Placebo (N=6 subjects)	
**Stage 1**	**Total**	**Grade ≥3**	**Total**	**Grade ≥3**
**Blood and lymphatic system disorders**				
Anemia	1 (16.7)	0	0	0
Neutropenia	0	0	1 (16.7)	1 (16.7)
**Gastrointestinal disorders**				
Nausea	3 (50.0)	0	4 (66.7)	1 (16.7)
Dry mouth	3 (50.0)	0	3 (50.0)	0
Oral pain	1 (16.7)	0	1 (16.7)	0
Constipation	2 (33.3)	0	2 (33.3)	0
Stomatitis	1 (16.7)	0	1 (16.7)	1 (16.7)
Diarrhea	1 (16.7)	0	3 (50.0)	0
Vomiting	1 (16.7)	0	2 (33.3)	1 (16.7)
Dysphagia	0	0	1 (16.7)	0
**General disorders**				
Fatigue	3 (50.0)	0	5 (83.3)	0
Weight loss	2 (33.3)	0	1(16.7)	1 (16.7)
**Infections and infestations**				
Oral candidiasis	1 (16.7)	0	1 (16.7)	0
**Injury, poisoning and procedural complications**				
Radiation skin injury	1 (16.7)	0	1 (16.7)	0
**Laboratory Investigations**				
Blood creatinine increased	1 (16.7)	0	1 (16.7)	0
**Metabolism and nutrition disorders**				

Dehydration	1 (16.7)	1 (16.7)	1 (16.7)	1 (16.7)
Decreased appetite	1 (16.7)	0	1 (16.7)	0
**Nervous system disorders**				
Dysgeusia	3 (50.0)	0	0	0
Taste disorder	1 (16.7)	0	2 (33.3)	0
**Respiratory, thoracic and mediastinal disorders**				
Oropharyngeal pain	1 (16.7)	0	3 (50.0)	0
**Preferred Term n (%)**	**EC-18 2000 mg (N=40 subjects)**		**Placebo (N=34 subjects)**	
**Stage 2**	**Total**	**Grade ≥3**	**Total**	**Grade ≥3**
**Blood and lymphatic system disorders**				
Anemia	5 (12.5)	1 (2.5)	3 (8.8)	0
**Ear and labyrinth disorders**				
Tinnitus	5 (12.5)	0	7 (20.6)	0
**Gastrointestinal disorders**				
Nausea	29 (72.5)	4 (10)	24 (70.6)	3 (8.8)
Dysphagia	16 (40.0)	5 (12.5)	15 (44.1)	0
Dry mouth	17 (42.5)	0	10 (29.4)	1 (2.9)
Vomiting	11 (27.5)	3 (7.5)	11 (32.4)	4 (11.8)
Stomatitis	13 (32.5)	7 (17.5)	8 (23.5)	1 (2.9)
Constipation	8 (20.0)	0	10 (29.4)	0
Oral pain	8 (20.0)	1 (2.5)	7 (20.6)	0
Diarrhea	5 (12.5)	1 (2.5)	6 (17.6)	0
**General disorders**				
Fatigue	17 (42.5)	0	12 (35.3)	1 (2.9)
Weight Loss	7(17.5)	0	10(29.4)	1 (2.9)
**Infections and infestations**				

Pharyngitis	6 (15.0)	0	3 (8.8)	0
**Injury, poisoning, and procedural complications**				
Radiation skin injury	9 (22.5)	1 (2.5)	9 (26.5)	0
**Laboratory Investigations**				
Blood creatinine increased	5 (12.5)	2 (5)	6 (17.6)	1 (2.9)
Platelet count decreased	5 (12.5)	0	3 (8.8)	0
White blood cell count decreased	5 (12.5)	4 (10)	3 (8.8)	1 (2.9)
**Nervous system disorders**				
Dysgeusia	15 (37.5)	0	14 (41.2)	0
**Metabolism and nutrition disorders**				
Dehydration	2 (5.0)	0	8 (23.5)	1 (2.9)
**Respiratory, thoracic, and mediastinal disorders**				
Oropharyngeal pain	7 (17.5)	0	6 (17.6)	0
Laryngeal inflammation	3 (7.5)	0	6 (17.6)	0

n = Number of subjects

Note: AEs were coded using MedDRA version 22.0. For each SOC and PT, subjects were included only once, even if they experienced multiple events in that system organ class or preferred term. TEAE was defined as an AE that first occurs or worsens in severity on or after the first dose of study drug through 30 days post last dose of study drug.

Table S3

AE n (%)	EC-18 2000 mg (N=46)	Placebo (N=40)
Fatigue	20 (43.5)	17 (42.5)
Tinnitus	5 (10.9)	7 (17.5)
Oral candidiasis	1 (2.2)	1 (2.5)
Pharyngitis	6 (13)	3 (7.5)
Laryngeal inflammation	3 (6.5)	6 (15)
Oropharyngeal pain	8 (17.4)	9 (22.5)
Dysgeusia	18 (39.1)	14 (35)
Taste disorder	1 (2.2)	2 (5)
Decreased appetite	1 (2.2)	1 (2.5)
Dehydration	3 (6.5)	9 (22.5)
Constipation	10 (21.7)	12 (30)
Diarrhea	6 (13)	9 (22.5)
Dry mouth	20 (43.5)	13 (32.5)
Dysphagia	16 (34.8)	16 (40)
Nausea	32 (69.6)	28 (70)
Oral pain	9 (19.6)	8 (20)
Stomatitis	14 (30.4)	9 (22.5)
Vomiting	12 (26.1)	13 (32.5)
Blood creatinine increased	6 (13)	7 (17.5)
Platelet count decreased	5 (10.9)	3 (7.5)
Weight decreased	9 (19.6)	11 (27.5)
White blood cell count decreased	5 (10.9)	3 (7.5)
Anemia	6 (13)	3 (7.5)

Neutropenia	0 (0)	1 (2.5)
Radiation skin injury	10 (21.7)	10 (25)

Table S4. Categorical Incidence of SAEs in EC-18 vs PL population

SAEs n (%)	EC-18 2000 mg (n=46)	Placebo (n=40)
Blood and lymphatic system disorders	0	1 (2.5)
Gastrointestinal Disorders	5 (10.9)	4 (10)
General disorders and administration site conditions	1 (2.2)	0
Infections and infestations	0	2 (5)
Injury, poisoning and procedural complications	0	1 (2.5)
Metabolism and nutrition disorders	0	3 (7.5)
Nervous system disorders	0	2 (5)
Renal and urinary disorders	1 (2.2)	4 (10)
Respiratory, thoracic and mediastinal disorders	1 (2.2)	1 (2.5)
Vascular disorders	0	1 (2.5)

Table S5. ITT Duration of SOM BL-LDRT all subjects

	Mean	Median
EC-18	2.8	0
PL	15.8	18
P value (t-test)	<0.001	n/a

Table S6. ITT Duration of SOM BL-LDRT in subjects who had ≥1 day of SOM

	Mean	Median
EC-18	9.3	8
PL	20.8	21
P value (t-test)	<0.001	n/a

Table S7: Subject Compliance

	Non-Compliant n (%)
EC-18 (n=36)	14 (38.9)
PL (n=32)	12 (37.5)
P value (chi-square)	0.9

Figure S1: Time to opioid analgesic use (PP Population)

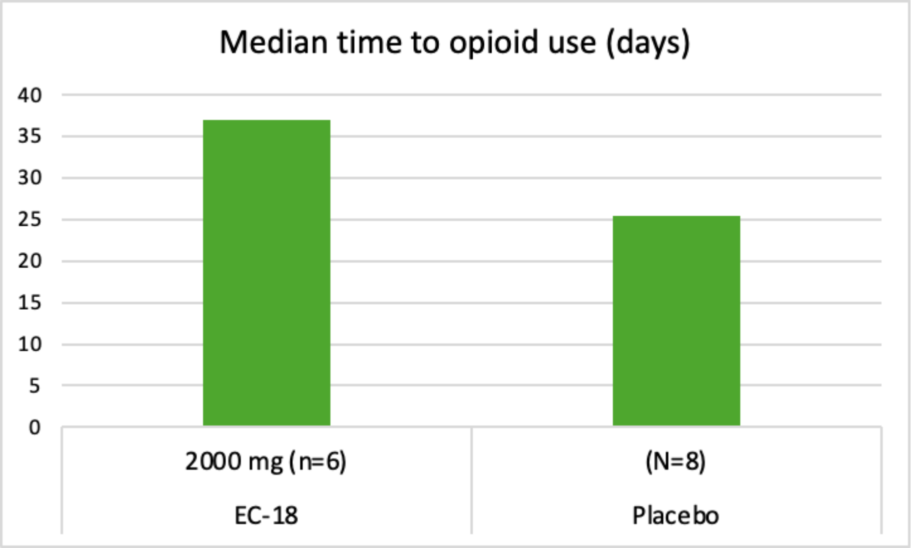

Supplement: Supplementary file 1 [file cancers-17-01663-s001.zip › cancers-3587780-SM.pdf]
